# Gastric Injury in Percutaneous Transhepatic Liver Biopsy After Living Donor Liver Transplantation: A Report of Two Cases

**DOI:** 10.7759/cureus.68789

**Published:** 2024-09-06

**Authors:** Takahiko Omameuda, Yukihiro Sanada, Yasunaru Sakuma, Yasuharu Onishi, Naohiro Sata

**Affiliations:** 1 Division of Gastroenterological, General and Transplant Surgery, Department of Surgery, Jichi Medical University, Shimotsuke, JPN

**Keywords:** gastric injury, gastrointestinal bleeding, living donor liver transplantation, percutaneous transhepatic liver biopsy, ultrasound-guided

## Abstract

Percutaneous transhepatic liver biopsy (PTLB) is essential for assessing liver function but carries risks such as bleeding, cholangitis, bowel injuries, and rare fatal complications. Gastric injury following PTLB is rare and not widely reported. This report describes two cases of gastric injury during ultrasound (US)-guided PTLB in patients following living donor liver transplantation. Gastric injury is uncommon, particularly when sampling from the left lobe due to its proximity to the stomach. Ensuring a clear field of vision, meticulous equipment preparation, and skilled technique are crucial for safe PTLB. When there is a risk of gastric injury, using smaller and shorter needles or alternative methods to US-guided PTLB is essential. Gastric injury should be promptly considered and treated if multiple punctures are required and if abdominal symptoms or gastrointestinal bleeding occur after PTLB.

## Introduction

Liver transplantation (LT) is a well-established treatment for irreversible acute and chronic liver diseases. Following LT, various complications can arise both in the early and late stages, making liver biopsy crucial for accurate diagnosis [1]. Percutaneous transhepatic liver biopsy (PTLB) is essential for diagnosing post-transplant complications such as acute cellular rejection, chronic rejection, recurrence of the original disease, steatosis, drug-induced hepatitis, infections, and other conditions. Despite its diagnostic utility, liver biopsy is an invasive procedure associated with a range of potential complications. Studies have reported a complication frequency of 0.2-5.9%, with pain and bleeding being the most common adverse events [2-5]. In the context of transplanted livers, post-biopsy complications can include cholangitis, sepsis, subcapsular hematoma, and arterioportal fistula [5].

Visceral injuries, although rare (0.01-0.1%), typically involve organs such as the gallbladder, colon, and right kidney [6]. When performing living donor liver transplantation (LDLT) in pediatric patients, the left lobe and lateral segment graft are often chosen, and the stomach is located adjacent to the graft. Notably, gastric injuries have not been documented in the literature.

This report presents two cases of gastric injury that occurred during ultrasound (US)-guided PTLB in patients who had undergone LDLT. These cases highlight the need for heightened awareness of such rare but serious complications.

## Case presentation

Case 1

**Figure 1:**
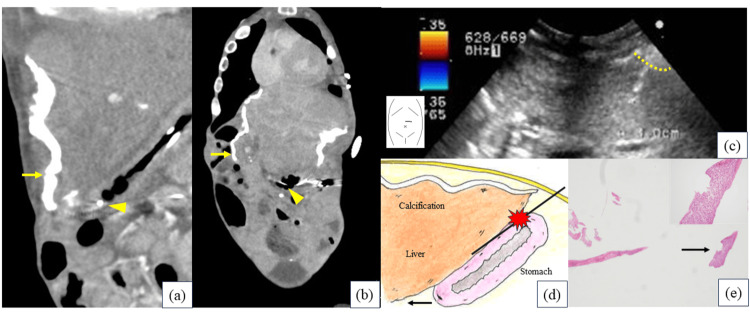
Images in Case 1. (a, b) Computed tomography (CT) findings: A CT scan shows that the transplanted liver (S2 monosegment graft) was thin and exhibited calcification on its surface (yellow arrow). The stomach was located caudal to the liver, and a nasogastric tube (yellow arrowhead) was inserted into the stomach. (a) Sagittal section. (b) Coronal section. (c) Ultrasonography findings: Ultrasonography shows the stomach above the transplanted liver close to the puncture site. The boundary (yellow dotted line) between the stomach and liver was somewhat unclear. Unaware of this, we punctured the stomach and the liver. (d) Schematic diagram: The schematic diagram shows the anatomical locations of the liver and stomach, with the black line representing the puncture line. Block arrow, cranial. Figure 1d was created by the authors. (e) Pathological findings: Hematoxylin and eosin staining reveals the presence of liver tissue and gastric mucosa (black arrow).

An eight-month-old boy after LDLT, who had received an S2 monosegment graft from his father for acute liver failure, underwent US-guided PTLB owing to liver dysfunction. A computed tomography (CT) scan showed calcification on the thin surface of the liver graft, which may be caused by ischemic injury after LT. The stomach was found to be in contact with the caudal side of the liver (Figures 1a, 1b). The first puncture was performed by selecting an area of very thin calcification, but it still failed to penetrate the calcification. Subsequently, a second puncture was performed. During the second puncture, we intentionally tilted the probe and performed the puncture to avoid calcification on the liver surface. However, we failed to recognize that the stomach above the transplanted liver was delineated close to the puncture site (Figures 1c, 1d). After the second puncture, a transient bloody discharge was observed from the nasogastric tube; however, this symptom improved after discontinuing tube feeding and administering a proton pump inhibitor (PPI) for one day. Blood test results showed no progression of anemia. Rapid pathological diagnosis revealed sampling of the gastric mucosa, indicating gastric injury during the PTLB (Figure 1e).

Case 2

**Figure 2:**
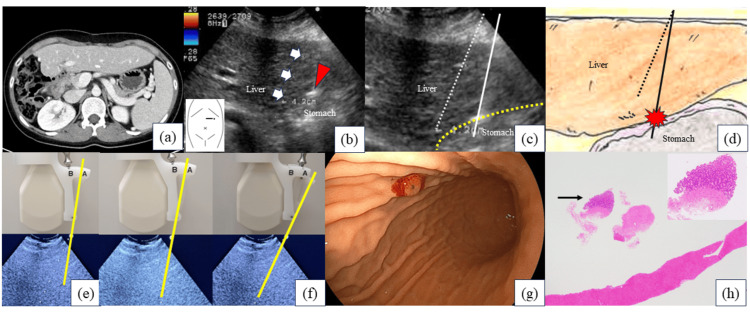
Images in Case 2. (a) CT scan findings: The CT scan reveals that the transplanted liver was thin in the left lateral segment, with the stomach positioned directly below it. (b, c) Ultrasonography findings: (b) Ultrasonography shows that the puncture line and monitor guide (white arrows) were incompatible. The red arrowhead indicates the site of the gastric injury. (c) The magnified image reveals the actual puncture line (white line), the monitor guide (white dotted line), and the boundary (yellow dotted line) between the stomach and liver. (d) Schematic diagram: The schematic diagram illustrated the anatomical positions of the liver and stomach, with the black line representing the actual puncture line and the dotted line representing the monitor guide. The block arrow indicates cranial. Figure 1d was created by the authors. (e, f) Ultrasonography findings: The angle of the actual puncture line (yellow line) was incompatible with the angle of the monitor guide (e). The angle of the actual puncture line (yellow line) was compatible with the angle of the monitor guide (f). (g) Esophagogastroduodenoscopy findings: A biopsy needle puncture mark can be observed on the anterior wall of the greater curvature of the gastric body. (h) Pathological findings: Hematoxylin and eosin staining reveals the presence of liver tissue and gastric mucosa (black arrow).

A 23-year-old woman who received an LDLT with a left lateral segment graft from her mother for biliary atresia underwent US-guided PTLB for protocol liver biopsy. The liver allograft was thin, and the stomach was located directly below it (Figure 2a). Moreover, we accidentally performed the puncture without realizing that the angle of the actual puncture line did not match that of the monitor guide (Figures 2b-2d). Moreover, we accidentally performed the puncture without realizing that the angle of the actual puncture line did not match that of the monitor guide (Figures 2b-2d). Figure 2b shows the incompatibility between the puncture line and the monitor guide. In particular, the magnified portion of Figure 2b, presented as Figure 2c, revealed the actual puncture line (white line), the monitor guide (white dotted line), and the boundary (yellow dotted line) between the stomach and liver. Additionally, Figure 2d provides a schematic diagram illustrating the anatomical locations of the liver and stomach, with the black line representing the actual puncture line and the dotted line representing the monitor guide. Therefore, the first puncture yielded an insufficient amount of tissue, so a second puncture was performed. During the second puncture, we noticed that the positioning of the puncture line did not align with that of the monitor guide (Figures 2e, 2f) and made the necessary corrections. The patient experienced nausea the following day; however, she was discharged as her condition improved. Melena was observed after discharge, with no improvement, prompting the patient to visit our hospital five days after the PTLB. Anemia (after the procedure: hemoglobin (Hb), 6.9 g/dL; before the procedure: Hb, 10.1 g/dL) was noted. During esophagogastroduodenoscopy for a closer examination, a biopsy needle puncture mark was found on the anterior wall of the greater curvature of the gastric body (Figure 2g). The patient’s symptoms improved with four units of red blood cell transfusion, PPI, and gastric mucosa-protective drugs, and she was discharged nine days after emergency admission. Pathological examination revealed gastric mucosal sampling, indicating gastric injury during the PTLB (Figure 2h).

## Discussion

In our department, all PTLBs were performed with the patient in the supine position once using a US-guided 16-gauge (G) Monopty needle (C. R. Bard, Inc., New Providence, NJ, USA), 22 mm in length, by transplant surgeons. If patients had an increased risk of bleeding or a small graft, an 18-G Monopty needle with a length of 11 mm was used. The needle approach was anterior, with the right lateral approach used for the left lobe or monosegment graft, and the right lobe or whole graft, respectively. Manual compression hemostasis was performed for 20 minutes with continuous monitoring of vital signs. Then, a round, hard weight made from gauze was applied with intermittent monitoring (three times per day) until the following day. After PTLB, patients rested in bed for two hours, then could walk for two hours, and were allowed to ambulate freely the following day [5].

First, during PTLB, sampling from the left lobe poses a higher risk of gastric injury due to its thinness and anatomical proximity to the stomach compared with the right lobe. Various risk factors have been reported to influence the complications of liver biopsy [6,7], including those related to puncture of other viscera, e.g., patient cooperation, operator experience, use of image guidance, type of technique (percutaneous/transvenous), and the number of needle passes. In addition to these risks, gastric injury should be considered, particularly when sampling from the thin left lobe. This applies not only to samples from transplanted livers, such as the left lobe and lateral segment, but also to the left lobe.

Second, preventive measures against gastric injury during liver biopsy should be considered. Although US-guided PTLB is reportedly safer than blind PTLB [8,9], gastric injury can occur even under US-guided conditions. In Case 1, the issue was that, in addition to limited visibility due to calcification, we failed to notice a portion of the stomach caudal to the liver allograft. In contrast, in Case 2, the angle of the actual puncture line and the monitor guide were incompatible, resulting in the puncturing of the stomach beneath the thin transplanted liver. A review of Case 1 shows that the puncture site must be clearly delineated and should only be punctured if it is clear that no other viscera are involved. Image-guided liver biopsies should be used for patients with altered liver anatomy, such as split liver graft or prior liver resection, and recent liver imaging (within the preceding three months) should be available [7]. We believe that, especially in cases with risks of gastric injury, a CT or MRI scan before PTLB is recommended to clarify the morphology of the liver and its anatomical relationship with adjacent viscera, as well as to identify the optimal puncture site if necessary. Based on this consideration, we could confirm the position where calcifications and the stomach caudal to the transplanted liver would not obstruct puncture using CT, and then delineate the optimal puncture position using ultrasonography. Then, PTLB was performed at one year and two years and one month. The optimal puncture site was identified, and a biopsy was successfully performed using a 16-G Monopty needle, 22 mm in length. Calcification on the surface of the liver graft may interfere with US-guided PTLB, and when calcification is observed, it may be particularly necessary to perform a detailed examination using CT or MRI before performing PTLB. Additionally, based on the review of Case 2, it is necessary to thoroughly check the equipment preparation before puncture. In general, to obtain an intact core with sufficient tissue, the current recommendation is for a biopsy of a minimum length of 20 mm, obtained with a 16-G needle [10,11]; however, we consider it advisable, for safety reasons, to prepare an 11-mm long needle in advance if the liver is thin and if other viscera are directly below it. Despite these precautions, if the condition does not guarantee a safe US-guided puncture position or the first puncture does not provide a sufficient amount of tissue, it would be acceptable to switch to transvenous US-guided liver biopsy [12], CT-guided liver biopsy, and laparotomy instead of US-guided PTLB.

Third, early action is important if other visceral injuries, including gastric injuries or bleeding, are suspected. The key to managing complications such as bleeding and visceral perforation is maintaining a high clinical suspicion. Suspicion of a potential complication should be high when the patient complains of pain that is disproportionate to the clinical events surrounding the biopsy and is associated with a decrease in blood pressure and tachycardia, which can be confirmed by radiological investigation [6]. In Case 1, bleeding in the stomach was detected by the bloody changes in the gastric tube drainage, whereas in Case 2, bleeding was detected through melena after discharge. In Case 2, bleeding could have been detected if blood samples had been taken at the time of nausea. When other visceral punctures are suspected, the progression of anemia should be monitored using blood tests. Additionally, in both cases, abdominal ultrasonography was performed immediately after the puncture and the following day, revealing no bleeding in the free abdominal cavity. Presumably, post-transplant patients have strong adhesions between the stomach and transplanted liver and are less likely to experience perforation or bleeding into the free abdominal cavity. However, careful investigation using imaging modalities, such as US and CT, is necessary if other visceral injuries are suspected. Patients should only be discharged if they are hemodynamically stable, with no evidence of bleeding, stable blood pressure, and no new complaints of pain or shortness of breath [13]. Furthermore, the pathology results in both cases revealed the presence of gastric mucosa, confirming that gastric injury occurred. This finding underscores the importance of ensuring that liver biopsy specimens are not contaminated with other tissues. Proper identification and evaluation of the specimen are crucial to distinguish between liver tissues and other tissues, thereby improving diagnostic accuracy and patient safety.

## Conclusions

Gastric injury, while rare, is a serious complication following liver biopsy, particularly when sampling from the left lobe due to its proximity to the stomach. In the cases presented, careful attention to ensuring a clear field of vision, meticulous equipment preparation, and skilled technique were key factors in minimizing this risk. The cases highlighted the importance of using smaller and shorter needles or considering alternative methods to US-guided PTLB when the risk of gastric injury is present. Additionally, the cases underscore the need for vigilance in monitoring gastric injury, especially in scenarios requiring multiple punctures or when patients develop abdominal symptoms or gastrointestinal bleeding after PTLB.
